# Effects of Prepubertal or Adult Site-Specific Knockdown of Estrogen Receptor β in the Medial Preoptic Area and Medial Amygdala on Social Behaviors in Male Mice^1^,^2^,^3^

**DOI:** 10.1523/ENEURO.0155-15.2016

**Published:** 2016-03-31

**Authors:** Mariko Nakata, Kazuhiro Sano, Sergei Musatov, Naoko Yamaguchi, Toshiro Sakamoto, Sonoko Ogawa

**Affiliations:** 1 Laboratory of Behavioral Neuroendocrinology, University of Tsukuba, Tsukuba, Ibaraki 305-8577, Japan; 2 Laboratory of Molecular Neurosurgery, Weill Cornell University Medical College, New York, New York 10021; 3 Department of Pharmacology, School of Medicine, Aichi Medical University, Nagakute, Aichi 480-1195, Japan; 4 Department of Psychology, Kyoto Tachibana University, Kyoto, Kyoto 607-8175, Japan

**Keywords:** aggressive behavior, estrogen receptor β, medial amygdale, medial preoptic area, sexual preference, site-specific knockdown

## Abstract

Testosterone, after being converted to estradiol in the brain, acts on estrogen receptors (ERα and ERβ) and controls the expression of male-type social behavior. Previous studies in male mice have revealed that ERα expressed in the medial preoptic area (MPOA) and medial amygdala (MeA) are differently involved in the regulation of sexual and aggressive behaviors by testosterone action at the time of testing in adult and/or on brain masculinization process during pubertal period. However, a role played by ERβ in these brain regions still remains unclear. Here we examined the effects of site-specific knockdown of ERβ (βERKD) in the MPOA and MeA on male social behaviors with the use of adeno-associated viral mediated RNA interference methods in ICR/Jcl mice. Prepubertal βERKD in the MPOA revealed that continuous suppression of ERβ gene expression throughout the pubertal period and adulthood decreased aggressive but not sexual behavior tested as adults. Because βERKD in the MPOA only in adulthood did not affect either sexual or aggressive behaviors, it was concluded that pubertal ERβ in the MPOA might have an essential role for the full expression of aggressive behavior in adulthood. On the other hand, although neither prepubertal nor adult βERKD in the MeA had any effects on sexual and aggressive behavior, βERKD in adulthood disrupted sexual preference of receptive females over nonreceptive females. Collectively, these results suggest that ERβ in the MPOA and MeA are involved in the regulation of male sexual and aggressive behavior in a manner substantially different from that of ERα.

## Significance Statement

We investigated the role played by estrogen receptor β (ERβ) expressed in the medial preoptic area (MPOA) and medial amygdala (MeA) in the regulation of male-type social behaviors with the use of RNA interference methods for brain site-specific ERβ knockdown (βERKD) in mice. We found that ERβ in the MPOA might be necessary for testosterone to fully masculinize the aggressive, but not sexual, behavior neural network through organizational action during the pubertal period. On the other hand, ERβ in the MeA may be involved in sexual information processing because βERKD male mice failed to show sexual preference toward a receptive female over a nonreceptive female. These finding are greatly contrasted with previously reported functions of ERα.

## Introduction

Gonadal steroid hormones play an essential role in the regulation of social behaviors. In male mice, testosterone is known to be indispensable for a series of male-type social behaviors. Testosterone is mainly secreted from the testes into the blood stream and binds either to androgen (AR) or estrogen (ER) receptors, after conversion to estradiol by aromatase in the brain.

Two subtypes of ERs, ERα and ERβ, are known to mediate intracellular actions by aromatized testosterone. Previous studies have reported that ERα and ERβ may play different roles in the regulation of male social behavior. ERα is necessary for induction, because systemic knock-out of ERα (αERKO) in male mice caused severe deficits in sexual and aggressive behaviors (Ogawa et al., 1997, 1998, 2000; Rissman et al., 1997; Wersinger et al., 1997). On the other hand, ERβ may play a modulatory role on the expression of these behaviors. It is reported that ERβ knock-out (βERKO) mice show increased levels of aggressive behavior depending on age and social experiences (Ogawa et al., 1999; Nomura et al., 2002, 2006), hyper-reactivity to social stimuli (Handa et al., 2012; Tsuda et al., 2014), altered risk-taking behavior (Kavaliers et al., 2008) and increased levels of anxiety (Walf and Frye, 2006; Weiser et al., 2008; Tomihara et al., 2009; Oyola et al., 2012).

ERα and ERβ are expressed in several brain regions in the limbic and hypothalamic regions (Shughrue et al., 1997; Mitra et al., 2003), which are main components of the social behavior neural network (Newman, 1999; Nelson and Trainor, 2007; Hull and Rodriguez-Manzo, 2009). Although there are brain areas expressing primarily ERα [eg, hypothalamic ventromedial nucleus (VMN)] or ERβ (eg, hypothalamic periventricular nucleus, midbrain dorsal raphe), a number of regions such as the medial preoptic area (MPOA) and medial amygdala (MeA) are known to express both types of ERs. In a recent study, achieved site-specific knockdown of ERα (αERKD) in adult (16 weeks of age) male mice using adeno-associated viral vector (AAV)-mediated RNA interference (RNAi), lack of ERα in the VMN reduced both of sexual and aggressive behaviors, whereas, ERα disruption in the MPOA decreased only sexual behavior and in the MeA failed to alter either behavior (Sano et al., 2013). On the other hand, when ERα was prepubertally knocked down in the MeA at postnatal day (P) 21 (which permanently suppressed ERα expression thereafter), both sexual and aggressive behaviors tested in adulthood were greatly reduced (Sano et al., 2015). These findings suggest that ERα in each site is differently involved in the regulation of sexual and aggressive behaviors, through either organizational action during pubertal period (Schulz et al., 2004; Sisk and Foster, 2004; Sisk, 2015) or activational action at the time of testing in adult.

In contrast to ERα, the brain site-specific role of ERβ for both organizational and activational action by gonadal steroids still remains unclear. Studies using an ERβ-specific agonist diarylpropionitrile (DPN) revealed that neonatal ERβ activation may play a role in the expression of male social behavior in adulthood (Patisaul and Bateman, 2008), but a role of ERβ during pubertal period has not been identified. Furthermore, in adulthood, it is reported that DPN treatment into the MeA facilitates male sexual behavior in gonadectomized rats only in males simultaneously treated with an ERα agonist (Russell et al., 2012). Considering that ERβ may be involved primarily in modulatory regulation of behavior, it is necessary to further investigate the effects of brain-site-specific manipulation of ERβ in animals that are otherwise maintained as intact. Among a number of brain sites expressing high levels of ERβ, we have focused on the MPOA and MeA to determine the effects of prepubertal and postpubertal knockdown. These two areas have been implicated in the regulation of male social behaviors including not only sexual and aggressive behavior (Hull et al., 1999; Paredes, 2003; Veening et al., 2005) but also male-type sexual preference (Kondo and Sachs, 2002; Dhungel et al., 2011) and social information processing (Baum, 2009).

In the present study, we examined the effects of prepubertal application of AAV-mediated ERβ silencing, which site-specifically disrupts the expression of ERβ (βERKD) both during pubertal period and at the time of testing in adult (Experiment 1). Given positive knockdown effects on behaviors, we then tested whether similar behavioral alteration could be induced in mice with postpubertal βERKD in the MPOA (Experiment 2). In the MeA where prepubertal βERKD had no effects on sexual and aggressive behaviors, we performed more thorough analysis of male-type sexual preference (Experiment 3).

## Materials and Methods

### Experimental animals

Gonadally intact ICR/Jcl male mice were used as experimental animals. They were originally purchased from a commercial breeder (CLEA Japan) and maintained in a breeding colony at the University of Tsukuba. All mice were kept under standard housing conditions (23±2°C, 12 h light/dark cycle with lights off at 12:00 P.M.) in polypropylene clear plastic cages (19×29×12 cm; Allentown) with corncob bedding. Food and water were provided *ad libitum.* All procedures were conducted in accordance with the National Institutes of Health guidelines and were approved by the Animal Care and Use Committee and the Recombinant DNA Use Committee at University of Tsukuba. All efforts were made to minimize the number of animals and their suffering.

### Estrogen receptor β silencing using small hairpin RNA

Experimental animals were stereotaxically injected with AAV vectors expressing a small hairpin RNA (shRNA), either prepubertally on P21 (Experiment 1) or postpubertally in adulthood (Experiments 2 and 3). AAV-shRNA against either the sequence specific for the ERβ gene (AAV-shERβ: 5′-GATCCCCGCCACGAATCAGTGTACCATCTTCCTGTCAATGGTACACTGATTCGTGGCTTTTTTGGAAT-3′ and 5′-CTAGAGCCACGAATCAGTGTACCATTGACAGGAAGATGGTACACTGATTCGTGGCGGG-3′) or the sequence specific for luciferase (LUC) as control (AAV-shLUC: 5′-GATCCCCCCGCTGGAGAGCAACTGCATCTTCCTGTCAATGCAGTTGCTCTCCAGCGGTTTTTGGAA-3′ and 5′-CTAGTTCCAAAAACCGCTGGA GAGCAACTGCATGAGCAACTGCATTGACAGGAAGATGCAGTTGCTCTCCAGCGGGGG-3′) were used. The nucleotides specific for ERβ and LUC are underlined. These vectors also expressed enhanced green fluorescent protein (GFP) as a reporter to visually detect transfected neurons.

Mice were anesthetized with sodium pentobarbital (60 mg/kg) and placed in a stereotaxic frame (David Kopf Instruments). A 26 G injection needle attached to a 10 µl Hamilton syringe was inserted by aiming for either the MeA or MPOA (coordinates were determined for each experiment separately). Each animal was bilaterally injected with 1 µl of either AAV-shERβ or AAV-shLUC (10^12^ packaged genomic particles, 0.5 µl/hemisphere) over 5 min. The needle was left in place for an additional 10 min following the end of the infusion.

### Behavioral tests

#### Sexual behavior test

Each experimental animal was tested for sexual behavior against a receptive female mouse in its home cage. Each trial was 30 min and conducted under red light illumination during the dark phase of the light/dark cycle. At the beginning of each trial, a hormonally primed ovariectomized (OVX) ICR/Jcl female mouse was introduced. To ensure high sexual receptivity, all females were subcutaneously injected with 10 µg estradiol benzoate in 0.1 ml sesame oil at 48 and 24 h, and 500 µg progesterone in 0.1 ml sesame oil at 4–6 h before testing. Each male was tested against a different female mouse in each of the repeated trials. The number of mounts and intromissions, and the latency to the first mount or intromission were recorded.

#### Aggressive behavior test

Aggressive behavior was assessed in a resident-intruder paradigm for 15 min under red light illumination during the dark phase of the light/dark cycle. At the beginning of the test, an age-matched gonadally intact ICR/Jcl male mouse (intruder) was introduced into a home cage of an experimental animal (resident). All intruder mice were olfactory bulbectomized (OBX) and group-housed (3–5 animals/cage). OBX was conducted to inhibit offensive aggression by intruders. Each resident mouse was tested against a different intruder mouse in each of the repeated aggression tests. An aggressive bout was defined as a series of behavioral interactions consisting of at least one of the following: chasing, boxing, tail rattling, wrestling, biting, and offensive lateral attack (often accompanied by biting). The number and cumulative duration of aggressive bouts were recorded. A maximum of 3 s could elapse between two aggressive bouts were considered as one aggressive bout. If the interval exceeded 3 s, the two bouts were scored as two separate aggressive bouts.

#### Sexual preference test

Each experimental mouse was tested for sexual preferences of a receptive female over a nonreceptive female (PTFF) and a receptive female over an intact male (PTFM). Each test was 15 min and conducted under white light illumination (26 lux) during dark phase of the light/dark cycle. The testing apparatus consisted of a white plastic testing cage (31×35×17) placed centrally in a white polyvinyl chloride box (46×51×25 cm). The test cage was covered with a clear acrylic board during tests and a video camera was placed 57 cm from the bottom of the testing cage. Clear sectoral Plexiglas cylinders (7 cm in radius, 16 cm in height) with 13 holes (6 mm diameter) near the bottom 3 cm of the cylinder (Mouse Cylinder SIOT3, O’Hara) were used to present opponent mice. Experimental mice were able to sniff olfactory cues from stimulus mice through perforated parts of the cylinders.

At least 2 d before testing, each experimental mouse was transferred to a testing cage with clean bedding and allowed to establish its own home territory. On the day of testing, they were first habituated to two empty cylinders for 1 h. The cylinders were placed at diagonal corners of the testing cage. At the beginning of the test, empty cylinders were removed and two cylinders with stimulus animals were placed at the same two diagonal corners. In PTFF, a hormonally primed (for detailed conditions, see Sexual behavior test) OVX C57BL/6J female mouse [receptive female (RF)] and an OVX C57BL/6J female mouse without hormonal priming [nonreceptive female (XF)] were used as stimuli. In PTFM, a RF and a gonadally intact C57BL/6J male (IM) mouse were used. After completion of each test, cylinders were thoroughly washed, wiped with 70% ethanol, and then air-dried.

Social investigation (SI) was defined as sniffing toward each stimulus animal through the holes of the cylinder. The cumulative duration of SI to each stimulus mouse was recorded separately. A maximum of 1 s could elapse between two SIs to be considered as one bout. If the interval exceeded 1 s, they were recorded as two bouts.

#### Quantitative analysis of behavioral data

All behavioral tests were recorded using digital video cameras. All video recordings were scored by an experimenter unaware of the animals’ experimental group, using a digital event recorder program (Recordia 1.0b, O’Hara).

Behavioral data from sexual and aggressive behavior tests was analyzed by a two-way ANOVA with repeated measurements for main effects of vector treatment, tests, and their interactions. The data from sexual preference tests was analyzed in each vector treatment group separately by a paired *t* test between two stimulus mice. All data were analyzed using the SPSS v21.0 (SPSS). Statistically significant differences were considered at *p* < 0.05. Superscript letters listed with *p*-values correspond to the statistical tests shown in Table 1.

### Histological analysis

#### Preparation of brain tissues for immunohistochemistry

After completion of the last behavioral tests, all experimental animals were deeply anesthetized with heparin-containing pentobarbital sodium solution (60 mg/kg body weight, i.p.). They were then perfused through the left cardiac ventricle with 40 ml of 100 mm phosphate buffered saline (PBS), pH 7.2, for blood removal, followed by 40 ml of 4% paraformaldehyde-containing 100 mm phosphate buffer (PB), pH 7.2, for fixation with the use of a peristaltic pump. Brains were removed and postfixed in the same fixative at 4°C for 24 h. After cryoprotection in 30% sucrose in 100 mm PB at 4°C, coronal sections (30 µm thickness) were prepared using a freezing microtome. Serial sections were collected in sets of four at 120 µm intervals, and stored in anti-freezing buffer (30% ethylene glycol and 30% glycerol in 0.05 m Tris-buffered saline (TBS), pH 7.2, at −20°C until use.

#### Immunohistochemistry

Freely floating sections were incubated in PBS containing 0.2% triton X (PBS-X) with 0.3% H_2_O_2_ for 20 min at room temperature (RT) for blocking. After washing, sections were pretreated with 5% bovine serum albumin in PBS-X (blocking buffer) for 2 h at RT. The sections were then incubated with goat polyclonal anti-GFP antiserum (1:5000; ab6673, Abcam) in blocking buffer for 1 night at 4°C. They were washed and incubated with biotinylated rabbit anti-goat secondary antiserum (1:250; Vector Laboratories) in blocking buffer for 2 h at RT. After washing, sections were reacted to avidin-biotin complex (Vectastain ABC Elite kit, Vector Laboratories) PBS for 1 h at RT, and washed. They were then incubated in 0.02% diaminobenzidine (DAB) and 0.003% H_2_O_2_ in PBS for 2 min, followed by wash with PBS. A few sections from each group were also processed for double-immunohistochemical staining for GFP and ERβ. Prior to immunohistochemistry for GFP, they were incubated with rabbit polyclonal ERβ antiserum (1:1000; Z8P, lot 10766190, Zymed Laboratories) for 3 d at 4°C followed by biotinylated goat anti-rabbit secondary antiserum (1:250; Vector Laboratories) for 2 h and visualized in 0.03% DAB, 0.15% NiNH_4_SO_4_, and 0.003% H_2_O_2_ in TBS for 12–14 min, followed by wash with TBS, pH 7.2.

All sections were mounted on gelatin-coated slides, air-dried, dehydrated through ascending series of ethanol, cleaned with xylene, and coverslipped with Permount (Fisher Scientific).

#### Analysis of immunopositive cells

Nine sections containing the MPOA (bregma 0.38 to −0.58) and nine sections containing the MeA (bregma −1.10 to −2.06) were selected for histological analysis of immunopositive cells for GFP. Each brain area was photographed with a digital camera mounted to a microscope (BZ-X710, KEYENCE). Spread of GFP-immunopositive cells was recorded for confirmation of AAV infection in the targeted area. We also selected three double-immunostained sections in the MPOA (bregma 0.02, −0.10, and −0.22) and in the MeA (bregma −1.82, −1.94, and −2.06) where most intensive ERβ expression were observed in the control groups. In these sections, we counted (3 mice per group) number of ERβ-immunopositive cells and double-labeled cells for ERβ and GFP in each side of the hemisphere within the targeted site. The data was analyzed in each section separately by a Welch’s *t* test between two vector treatment groups using the SPSS v21.0 (SPSS). Statistically significant differences were considered at *p* < 0.05. Superscript letters listed with *p*-values correspond to the statistical tests shown in Table 1.

### Experimental Design

#### Experiment 1 : prepubertal treatment in the MPOA and MeA

**Figure 1.**
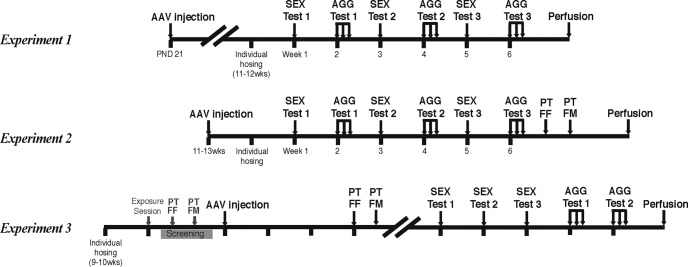
Experimental procedures of Experiment 1 (top), Experiment 2 (middle), and Experiment 3 (bottom). Ticks under the horizontal bar indicate 1 week. SEX, Sexual behavior; AGG, aggressive behavior.

A total of 12 litters of ICR/Jcl male mice were assigned to either MPOA or MeA groups on P21 after being weaned (Fig. 1, top). Mice from each litter were further subdivided into two shRNA injection groups of either AAV-shERβ or AAV-shLUC. Those four groups were designated as prepubertal treatment MPOA-βERKD (*n*=11), MPOA-Cont (*n*=13), MeA-βERKD (*n*=9), and MeA-Cont (*n*=9). Coordinates for the MPOA group were AP +0.02, ML ±0.5, DV −5.2, and those for the MeA group were AP −1.25, ML ±2.2, DV −5.15. All coordinates were determined based on The Mouse Brain Stereotaxic Coordinates (Paxinos and Franklin, 2001) with an adjustment for the brain size on P21. All mice were then group housed with their littermates (4–5 mice per cage) until they were tested for sexual and aggressive behavior in adulthood as gonadally intact (11.9±0.21 weeks of age at the first behavioral test). One week before the first behavioral test, all mice were individually housed. Three sexual behavior tests and three sets of aggressive behavior tests (each set consisting of aggression tests in 3 consecutive days) were done biweekly in alternate weeks for a total of 6 weeks. After the completion of the last behavioral test, brain tissues were collected and processed for immunohistochemistry for GFP and ERβ.

#### Experiment 2: postpubertal treatment in the MPOA

Gonadally intact adult male mice (12.2±1.00 weeks of age at injection) were stereotaxically injected with either AAV-shERβ (MPOA-βERKD, *n*=11) or AAV-shLUC (MPOA-Cont, *n*=14; Fig. 1, middle). Coordinates were AP +0.02, ML ±0.5, DV −5.65. One week after surgery, all mice were individually housed and a series of biweekly sexual and aggressive behavior tests (described in Experiment 1) was started on the following week. After the completion of behavioral tests, brain tissues were collected and processed for immunohistochemistry for GFP.

#### Experiment 3: postpubertal treatment in the MeA

Gonadally intact adult male mice were individually housed (9.7±0.49 weeks of age; Fig. 1, bottom). Starting 1 week later, they were given an exposure session. Briefly, a hormonally primed receptive C57BL/6J female mouse was placed in a clear columnar Plexiglas cylinder (7 cm in diameter, 16 cm in height with 28 holes of 6 mm diameter near the bottom 3 cm of the cylinder; Mouse Cylinder SIOT1, O’Hara) and presented in the center of the male’s home cage for 30 min. Starting at least 4 d after the exposure session, experimental animals were transferred to white plastic testing cages and given two screening sexual preference tests, one with PTFF and the other with PTFM paradigms. Only the mice that showed longer SI toward a receptive female over nonreceptive female (PTFF paradigm) and intact male (PTFM paradigm) were selected. They were then injected with either AAV-shERβ (MeA-βERKD, *n*=15), or AAV-shLUC (MeA-Cont, *n*=13) on 15–16 d after the completion of the screening tests. Coordinates: AP −1.7, ML ±2.4, DV −5.4. Three weeks after injections, all mice were given PTFF and PTFM sexual preference tests at 4 d intervals. Starting 1 week after the completion of sexual preference tests, they were given three weekly sexual behavior tests followed by two sets of aggressive behavior tests (each set consisting of aggression tests in 3 consecutive days) during 5 weeks. After the completion of the last behavioral test, brain tissues were collected and processed for immunohistochemistry for GFP.

## Results

### Experiment 1: prepubertal treatment in the MPOA and MeA

#### Effects of prepubertal silencing of ERβ in the MPOA on sexual and aggressive behavior

**Figure 2.**
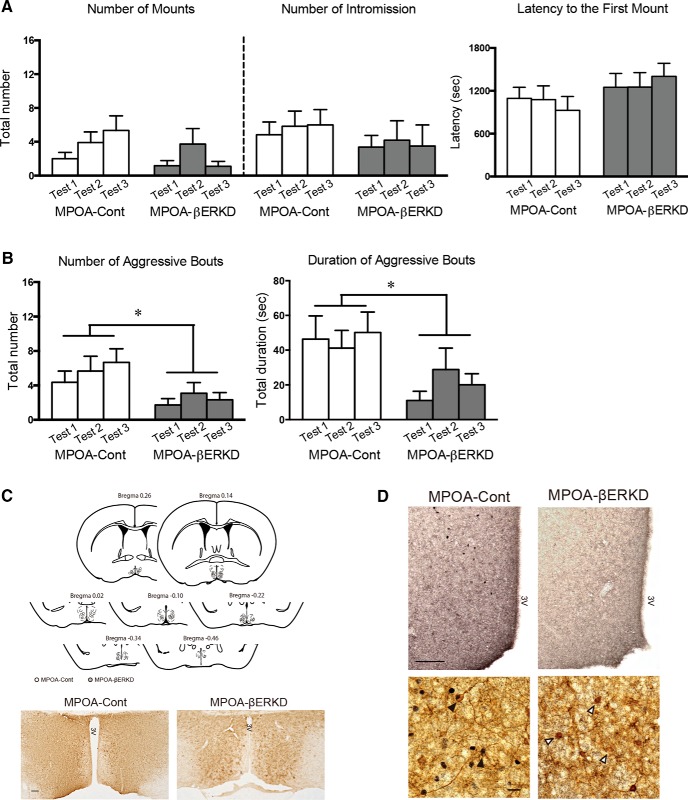
Effects of prepubertal silencing of ERβ in the MPOA on the expression of male sexual and aggressive behaviors in adulthood. ***A***, There was no difference between the MPOA-Cont and MPOA-βERKD groups in the number of mounts (left), intromissions (middle), or latency to the first mount (right). ***B***, The duration (left) and number (right) of aggressive bouts was significantly reduced in the MPOA-βERKD group compared with the MPOA-Cont group (*p< 0.05). All behavioral data in ***A*** and ***B*** are presented as mean + SEM. ***C***, Histological diagrams depicting the placement of the injection needle tip for each mouse in the MPOA-Cont (open circles) and MPOA-βERKD (solid circles) groups (top), and representative photomicrographs of MPOA sections with single-immunohistochemical staining for GFP (bottom; at bregma −0.10). Scale bar, 100 µm. 3V, third ventricle. ***D***, Representative photomicrographs of MPOA sections with single-immunohistochemical staining for ERβ (top; at bregma −0.22), and MPOA sections with double-immunostaining for GFP and ERβ (bottom). Number of ERβ-immunoreactive cells in the targeted site was reduced in the βERKD group compared with the control group. Scale bars: top, 100 µm; bottom, 20 µm. Bottom, Black arrowheads indicate ERβ and GFP double-immunoreactive cells and white arrowheads indicate immunoreactive cells only for GFP.

Prepubertal βERKD in the MPOA did not affect the expression of sexual behavior tested in adulthood (Fig. 2*A*). There were no significant main effects of treatment and test, and interaction of treatment and test in the number of mounts^a^ and intromissions^b^, or latency to the first mount^c^.

**Table 1.** Statistical table

	Data structure	Test	Exact *p* value	*N*
E1-MPOA				
a Mount *n*	Two-factor, mixed design: bw (trt) and wi (test)	ANOVA	0.305 (trt)0.086 (test)0.185 (trt × test)	10 KD; 10 Cont
b Intromission *n*	Two-factor, mixed design: bw(trt) and wi (test)	ANOVA	0.537 (trt)0.971 (test)0.741 (trt × test)	10 KD; 10 Cont
c Mount latency	Two-factor, mixed design: bw(trt) and wi (test)	ANOVA	0.366 (trt)0.925(test)0.130 (trt × test)	10 KD; 10 Cont
d Agg *n*	Two-factor, mixed design: bw(trt) and wi (test)	ANOVA	0.043 (trt)0.123 (test)0.434 (trt × test)	11 KD; 13 Cont
e Agg duration	Two-factor, mixed design: bw(trt) and wi (test)	ANOVA	0.035 (trt)0.544 (test) 0.203 (trt × test)	11 KD; 13 Cont
f cell *n*	One-factor, bw (trt)	Welch’s *t* test	0.045	6^+^ KD; 6^+^ Cont
g cell *n*	One-factor, bw (trt)	Welch’s *t* test	0.007	6^+^ KD; 6^+^ Cont
h cell *n*	One-factor, bw (trt)	Welch’s *t* test	0.007	6^+^ KD; 6^+^ Cont
E1-MeA				
i Mount *n*	Two-factor, mixed design: bw(trt) and wi (test)	ANOVA	0.677 (trt) 0.102 (test)0.919 (trt × test)	8 KD; 9 Cont
j Intromission *n*	Two-factor, mixed design: bw(trt) and wi (test)	ANOVA	0.285 (trt) 0.171 (test)0.069 (trt × test)	8 KD; 9 Cont
k Mount latency	Two-factor, mixed design: bw(trt) and wi (test)	ANOVA	0.972 (trt) 0.383 (test)0.600 (trt × test)	8 KD; 9 Cont
l Agg *n*	Two-factor, mixed design: bw(trt) and wi (test)	ANOVA	0.823 (trt) 0.246 (test)0.947 (trt × test)	9 KD; 9 Cont
m Agg duration	Two-factor, mixed design: bw(trt) and wi (test)	ANOVA	0.636 (trt) 0.815 (test)0.570 (trt × test)	9 KD; 9 Cont
n cell *n*	One-factor, bw (trt)	Welch’s *t* test	0.0001	6^+^ KD; 6^+^ Cont
o cell *n*	One-factor, bw (trt)	Welch’s *t* test	0.00001	6^+^ KD; 6^+^ Cont
p cell *n*	One-factor, bw (trt)	Welch’s *t* test	0.0009	6^+^ KD; 6^+^ Cont
E2				
q Mount *N*	Two-factor, mixed design: bw(trt) and wi (test)	ANOVA	0.069 (trt) 0.014 (test)0.202 (trt × test)	11 KD; 14 Cont
r Intromission *n*	Two-factor, mixed design: bw(trt) and wi (test)	ANOVA	0.123 (trt) 0.035 (test)0.228 (trt × test)	11 KD; 14 Cont
s Mount latency	Two-factor, mixed design: bw(trt) and wi (test)	ANOVA	0.077 (trt) <0.001 (test)0.153 (trt × test)	11 KD; 14 Cont
t Agg *n*	Two-factor, mixed design: bw(trt) and wi (test)	ANOVA	0.858 (trt) 0.021 (test)0.828 (trt × test)	11 KD; 14 Cont
u Agg duration	Two-factor, mixed design: bw(trt) and wi (test)	ANOVA	0.927 (trt) 0.036 (test)0.796 (trt × test)	11 KD; 14 Cont
E3				
v PTFF (Cont)	One-factor, wi (stim)	Paired *t* test	0.028	13
w PTFF (KD)	One-factor, wi (stim)	Paired *t* test	0.854	15
x PTFM (Cont)	One-factor, wi (stim)	Paired *t* test	0.001	13
y PTFM (KD)	One-factor, wi (stim)	Paired *t* test	<0.001	15
z Mount *n*	Two-factor, mixed design: bw(trt) and wi (test)	ANOVA	0.380 (trt) 0.001 (test)0.359 (trt × test)	14 KD; 12 Cont
aa Intromission *n*	Two-factor, mixed design: bw(trt) and wi (test)	ANOVA	0.608 (trt) <0.001 (test)0.694 (trt × test)	14 KD; 12 Cont
ab Mount latency	Two-factor, mixed design: bw (trt) and wi (test)	ANOVA	0.813 (trt) 0.001 (test) 0.437 (trt × test)	14 KD; 12 Cont
ac Agg *n*	Two-factor, mixed design: bw(trt) and wi (test)	ANOVA	0.262 (trt) 0.994 (test)0.884 (trt × test)	14 KD; 13 Cont
ad Agg duration	Two-factor, mixed design: bw(trt) and wi (test)	ANOVA	0.291 (trt) 0.040 (test)0.418 (trt × test)	14 KD; 13 Cont

*n*, Number; agg, aggressive bout; cell *n*, total number of ERβ-immunopositive cells; bw, between; wi, within; trt, vector treatment; KD, βERKD; Cont, Control; +, 2 hemispheres × 3 mice.

On the other hand, the levels of aggressive behavior were significantly reduced by prepubertal ERβ knockdown in the MPOA (Fig. 2*B*). Mice in the MPOA-βERKD group showed significantly fewer number (*F*_(1,22)_ = 4.631; *p* = 0.043^d^) and shorter duration (*F*_(1,22)_ = 5.078; *p* = 0.035^e^) of aggressive bouts compared with those in the MPOA-Cont group (main effects of test and interaction of treatment and test, n.s.^d,e^) . We also examined whether any specific component(s) of aggressive behavior, particularly lateral attacks, which are the most vigorous type of aggressive behavior, might be altered by the treatment. However, we did not find any specific effects of prepubertal ERβ manipulation in the MPOA.

Examination of placement of the injection needle tip (Fig. 2*C*, top) and presence of GFP-immunopositive cells confirmed successful bilateral injections of AAV vectors within the MPOA for all mice used in behavioral analysis (Fig. 2*C*, bottom). In addition, ERβ expression was examined immunohistochemically. The number of ERβ-immunoreactive cells in the MPOA was significantly reduced in the MPOA-βERKD group compared with those in the MPOA-Cont group (bregma +0.02, *t*_(6.789)_ = 2.449; *p* = 0.045^f^, bregma − 0.10, *t*_(5.147)_ = 4.315; *p* = 0.007^g^, bregma −0.22, *t*_(5.672)_ = 4.171; *p* = 0.007^h^, Fig. 2*D*, top; Table 2). Furthermore, coexpression of ERβ in GFP-immunopositive cells was detected by double-labeled immunohistochemistry in AAV-shLUC-injected control mice. On the other hand, ERβ expression was absent in the GFP-immunopositive cells of an AAV-shERβ-injected mice, although we found ERβ expression in a few GFP-negative cells in these mice (Fig. 2*D*, bottom; Table 2). These anatomical analysis confirmed successful knockdown of ERβ expression in transfected cells in the MPOA-βERKD group.

**Table 2.** Number of ERβ-immunopositive cells

		Control			βERKD		
		Total ERβ	Double-labeled		Total ERβ	Double-labeled	
	Bregma	Cell number	Cell number	% in total	Cell number	Cell number	% in total
MPOA	+0.02	20.5 ± 4.7	15.3 ± 3.6	76.2 ± 6.3	8.0 ± 2.0^*^	0	0
	-0.10	40.8 ± 8.2	32.0 ± 6.3	79.5 ± 3.8	5.0 ± 1.0^**^	0	0
	-0.22	9.8 ± 2.1	7.7 ± 2.1	74.2 ± 5.9	0.8 ± 0.5^**^	0	0
MeA	-1.82	158.8 ± 14.7	117.0 ± 9.0	73.9 ± 1.3	15.3 ± 4.0^**^	0.7 ± 0.3	3.6 ± 1.8
	-1.94	223.0 ± 19.1	141.8 ± 16.7	63.2 ± 4.8	80.8 ± 19.1^**^	1.3 ± 1.3	1.2 ± 1.2
	-2.06	198.7 ± 18.4	210.0 ± 15.5	70.1 ± 1.4	96.3 ± 11.5^**^	0.7 ± 0.3	0.8 ± 0.4

Total ERβ cell number, Number of ERβ-immunopositive cells/side in the targeted area; double-labeled cell number, number of double-stained cells with ERβ and GFP; double-labeled % in total, percentage of double-stained cells in the total number of ERβ-immunopositive cells.

*p< 0.05, **p< 0.01 versus Control.

#### Effects of prepubertal silencing of ERβ in the MeA on sexual and aggressive behaviors

**Figure 3.**
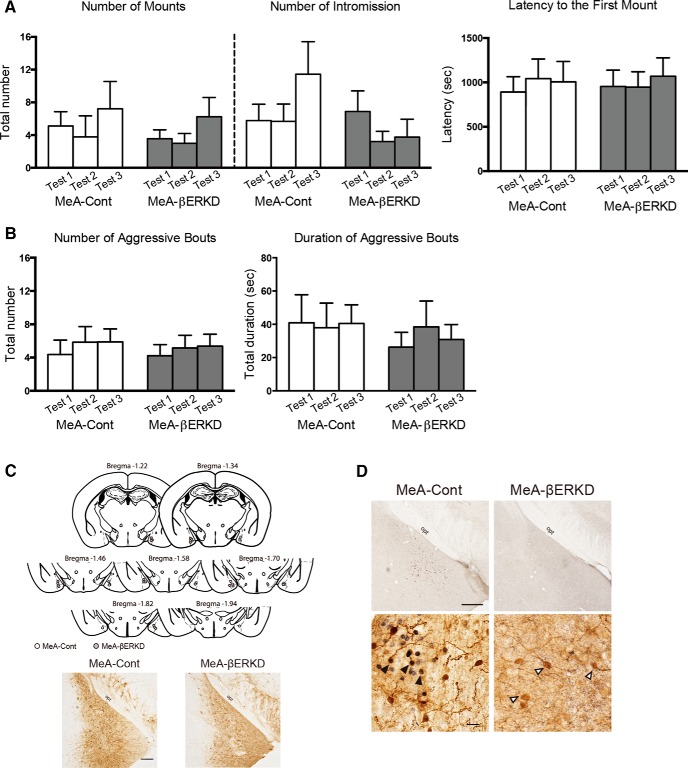
Effects of prepubertal silencing of ERβ in the MeA on the expression of male sexual and aggressive behaviors in adulthood. ***A***, There were no differences between the MeA-Cont and MeA-βERKD groups in the number of mounts (left), intromissions (middle), or latency to the first mount (right). ***B***, There were no differences between the MeA-Cont and MeA-βERKD groups in the duration (left) or number (right) of aggressive bouts. All behavioral data in ***A*** and ***B*** are presented as mean + SEM. ***C***, Histological diagrams depicting the placement of the injection needle tip for each mouse in the MeA-Cont (open circles) and MeA-βERKD (solid circles) groups (top), and representative photomicrographs of MeA sections with single-immunohistochemical staining for GFP (bottom ; at bregma −1.82). Scale bar, 200 µm. opt, optic tract. ***D***, Representative photomicrographs of MeA sections with single-immunohistochemical staining for ERβ (top; at bregma −1.94), and MeA sections with double-immunostaining for GFP and ERβ (bottom). Number of ERβ-immunoreactive cells in the targeted site was greatly reduced in the βERKD group compared with the control group. Scale bars: top, 200 µm; bottom, 20 µm. Bottom, Black arrowheads indicate ERβ and GFP double-immunoreactive cells and white arrowheads indicate immunoreactive cells only for GFP.

Prepubertal silencing of ERβ in the MeA had no effects on the expression of either sexual or aggressive behaviors in adulthood. The number of mounts^i^ and intromissions^j^, and latency to the first mount^k^ in the MeA-βERKD group were not different from those in the MeA-Cont group (Fig. 3*A*). Likewise, both number^l^ and duration^m^ of aggressive bouts were not different between the two treatment groups (Fig. 3*B*).

Examination of placement of the injection needle tip (Fig. 3*C*, top) and presence of GFP-immunopositive cells confirmed successful bilateral injections of AAV vectors within the MeA for all mice used in behavioral analysis (Fig. 3*C*, bottom). In addition, the number of ERβ-immunoreactive cells in the MeA was greatly reduced in the MeA-βERKD group compared with those in the MeA-Cont group (bregma −1.82, *t*_(5.739)_ = 9.443; *p* < 0.0001^n^, bregma −1.94, *t*_(7.485)_ = 5.267; *p* < 0.0001^°^, bregma −2.06, *t*_(8.407)_ = 9.314; *p* = 0.0009^p^, Fig. 3*D*, top; Table 2). Furthermore, double-labeled immunohistochemistry revealed that coexpression of ERβ and GFP was frequently observed in the AAV-shLUC-injected mice but not in the AAV-shERβ-injected mice (Fig. 3*D*, bottom; Table 2). These anatomical analysis confirmed successful knockdown of ERβ expression in transfected cells in the MeA-βERKD group.

### Experiment 2: postpubertal treatment in the MPOA

**Figure 4.**
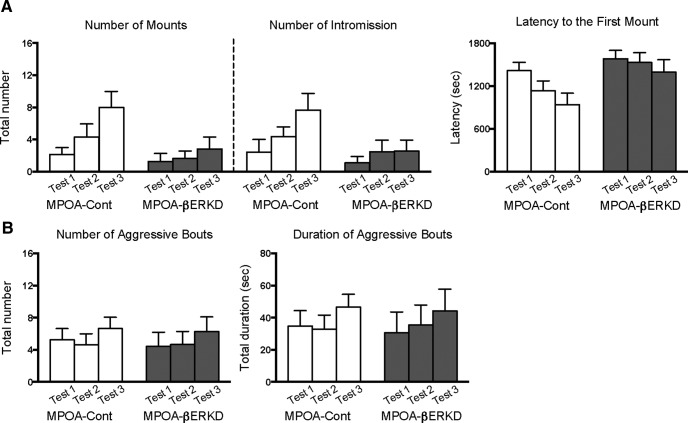
Effects of βERKD in the MPOA in adulthood on male sexual and aggressive behaviors. ***A***, There was no difference between the MPOA-Cont and MPOA-βERKD groups in the number of mounts (left), intromissions (middle), or latency to the first mount (right). ***B***, There was no difference between the MPOA-Cont and MPOA-βERKD groups in the duration (left) or number (right) of aggressive bouts. All behavioral data are presented as mean + SEM.

There was no difference in male sexual behaviors between adult MPOA-βERKD and MPOA-Cont groups (Fig. 4*A*), as predicted from the results of prepubertal βERKD in which ERβ expression was presumably suppressed throughout the life after AAV-shERβ injection (Experiment 1). There was no overall significant main effect of treatment nor interaction between treatment and test in the number of mounts^q^ and intromissions^r^ and the latency to the first mount^s^, although overall significant main effect of test (number of mounts: *F*_(1.712,39.373)_ = 5.078, *p* = 0.014^q^; number of intromissions: *F*_(1.448,33.296)_ = 4.185, *p* = 0.035^r^; both adjusted by Greenhouse–Geisser; latency to the first mount: *F*_(2,46)_ = 9.470, *p* < 0.001^s^).

On the other hand, effects of AAV-shERβ injection in adulthood on aggressive behavior greatly contrasted with those induced by prepubertal injection. Unlike suppressive effects on the number and duration of aggressive bouts by prepubertal βERKD found in Experiment 1, mice from MPOA-βERKD and MPOA-Cont groups showed equivalent levels of aggressive behaviors (Fig. 4*B*). There were no significant main effects of treatment and interaction of treatment and test on either measurement,^t,u^ although there were overall significant increases in the number (*F*_(2,46)_ = 4.199, *p* = 0.021^t^) and duration (*F*_(2,46)_ = 3.582, *p* = 0.036^u^) of aggressive bouts along repeated tests.

Examination of placement of the injection needle tip and presence of GFP-immunopositive cells confirmed successful bilateral injections of AAV vectors within the MPOA for all mice used in behavioral analysis (data not shown).

### Experiment 3: postpubertal treatment in the MeA

**Figure 5.**
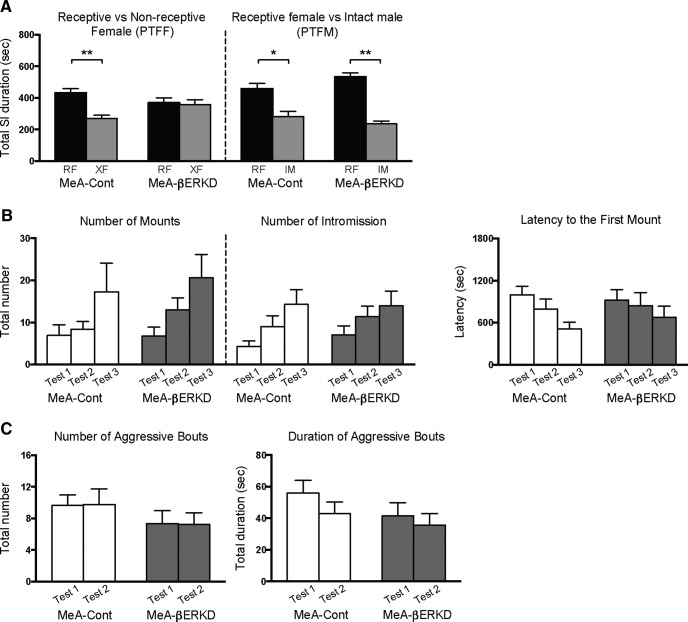
Effects of βERKD in the MeA in adulthood on male sexual preference and sexual and aggressive behaviors. ***A***, In PTFF tests, mice in the MeA-Cont group showed longer SI duration toward a receptive female (vs toward a nonreceptive female mouse) but mice in the MeA-βERKD group failed to do so (left). Both of the MeA-Cont and MeA-βERKD groups showed longer SI duration toward a receptive female in PTFM tests (vs toward a male mouse; right; *p< 0.05, **p< 0.01). ***B***, There were no differences between the MeA-Cont and MeA-βERKD groups in the number of mounts (left), intromissions (middle), or latency to the first mount (right). ***C***, There were no differences between the MeA-Cont and MeA-βERKD groups in the duration (left) or number (right) of aggressive bouts. All behavioral data are presented as mean + SEM.

For thorough analysis of sexual preference (which has been reported to be influenced by social experience), we performed two types of tests prior to sexual and aggressive tests in this experiment. We found that lack of ERβ in the MeA interferes with sexual preference toward a RF over a XF, tested in the PTFF paradigm but not over a IM, tested in the PTFM paradigm (Fig. 5*A*). In PTFF tests (Fig. 5*A*, left), MeA-Cont males investigated RFs significantly longer than XFs (*t*_(12)_ = 2.504, *p* = 0.028^v^), whereas MeA-βERKD males failed to show such preference (*t*_(14)_ = 0.199, *p* = 0.854, n.s.^w^). On the other hand, in PTFM tests (Fig. 5*A*, right), mice in both MeA-βERKD and MeA-Cont groups showed significantly longer SI duration toward RFs than toward IMs (βERKD: *t*_(14)_ = 7.446, *p* < 0.001^y^; Cont: *t*_(12)_ = 4.534, *p* = 0.001^x^).

ERβ silencing only in adulthood by postpubertal injection of AAV-shERβ had no significant effects on the expression of either sexual and aggressive behaviors, as predicted from the findings in prepubertal treatment in the MeA (Experiment 1). There were no overall significant differences between MeA-βERKD and MeA-Cont groups in the number of mounts^z^ and intromissions^aa^ or latency to the first mount^ab^ (Fig. 5*B*). In both groups, the levels of sexual behavior similarly increased with repetition of tests (number of mounts: *F*_(2,48)_ = 7.780, *p* = 0.001^z^; number of intromissions: *F*_(2,48)_ = 9.112, *p* < 0.001^aa^), with a decrease of latency to the first mount (*F*_(2,48)_ = 7.993; *p* = 0.001^ab^). In aggression tests, mice from MeA-βERKD and MeA-Cont groups showed similar levels of aggressive behavior in terms of both number^ac^ and duration^ad^ of aggressive bouts (Fig. 5*C*). Examination of placement of the injection needle tip and presence of GFP-immunopositive cells confirmed successful bilateral injections of AAV vectors within the MeA for all mice used in behavioral analysis (data not shown).

## Discussion

In the present study, we conducted site-specific knockdown of ERβ targeting the MPOA or MeA either on prepubertal (21 d old) or postpubertal (11 weeks old or later) age. We have found that ERβ in the MPOA during pubertal period may contribute to full expression of aggressive behavior in adulthood. In the MeA, we have found that ERβ may be specifically involved in the control of sexual preference. To confirm successful silencing of ERβ expression in AAV transfected cells in the targeted brain site, we have performed double-immunohistochemical staining and found all the cells stained for GFP were ERβ negative in βERKD groups. It should be noted that the specificity of commercially available ERβ antibodies except a few original lots of Zymed antibody Z8P has been controversial (Snyder et al., 2010). Therefore, we used an aliquot from one of earlier lots proven for its specificity previously (Shughrue and Merchenthaler, 2001; Nomura et al., 2003, 2005). Together, we could provide the first direct demonstration of site-specific role of ERβ in the regulation of male-type social behavior.

### Role of ERβ in the MPOA in the regulation of sexual and aggressive behavior

It has been known that the MPOA plays a role for the expression of male sexual behavior because copulation activates the MPOA neuronal activity (Coolen et al., 1997; Veening et al., 2005) and lesions of this area disrupt male sexual behavior (Hull et al., 2002). In the present study, any components of sexual behavior, ie, the number of mounts or intromissions, and the latency to the first mount, were affected by neither prepubertal nor postpubertal injection of AAV-shERβ to the MPOA. These results are contrasted with the findings reported in mice site-specifically knocked down for ERα in the MPOA (Sano et al., 2013, 2015). In these studies, both prepubertal and postpubertal injection of AAV-shERα in the MPOA significantly decreased the number of attempted mounts, mounts, and intromissions. Therefore, even though both ERα and ERβ are expressed in the MPOA (Shughrue et al., 1997; Mitra et al., 2003), it is concluded that ERα may be primarily responsible for the facilitation of sexual behavior. Our results also indicate that for facilitation of sexual behavior via ERα, simultaneous ERβ activation is not necessary. This is consistent with the finding reported in male rats treated with either ERα or ERβ-specific agonist site-specifically in the MPOA (Russell et al., 2012).

We found that duration and number of bouts of aggressive behavior were greatly reduced by AAV-shERβ injection into the MPOA on 21 d of age (Experiment 1), which permanently suppressed ERβ expression throughout the life after the treatment in the target area. On the other hand, similar treatment in 11 to 12 weeks of age did not affect either measurements of aggressive behavior (Experiment 2). Therefore, decreased levels of aggression found in Experiment 1 was not due to a lack of ERβ expression at the time of testing in adult. Rather, our data is interpreted that ERβ expression in the MPOA may be necessary for organizational action of testosterone during development, particularly during pubertal period. It may be argued that behavioral alteration caused by prepubertal ERβ knockdown in the MPOA might be due to reduced levels of testosterone in βERKD mice. Although we did not measure circulating levels of testosterone in the present study, this seems unlikely because global βERKO male mice are known to have similar or increased levels of testosterone in adult or during pubertal period compared to wild-type mice (Couse and Korach, 1999; Nomura et al., 2002). Previous studies have implicated involvement of ERβ in neonatal masculinization (Patisaul and Bateman, 2008) or defeminization (Kudwa et al., 2005) processes for the regulation of male sexual behavior. The present finding provides evidence that ERβ may also play a role in masculinization of the neural network of aggressive behavior and its site-specificity. Furthermore, an increased number of studies have now shown the importance of pubertal period for brain organization, in addition to classically known perinatal period (Schulz et al., 2004; Sisk and Foster, 2004; Sisk, 2015). A recent study has reported that ERα in the MeA may play a role in masculinizing action of testosterone during pubertal period (Sano et al., 2015). In this study, it is also reported that ERα expression in GFP-immunopositive cells at the target brain site was silenced by 5 d after AAV injection on P21. Because we used vectors with the same conformation, it is likely that ERβ expression was successfully reduced before the mice reached pubertal onset. Together, the present finding is the first demonstration indicating that ERβ may also participate in pubertal organizational action of testosterone. Molecular mechanisms of ERβ-mediated organizational action of testosterone, however, remains to be elucidated in future studies.

In male mice that reached adult age, injection of AAV-shRNA in the MPOA to knockdown ERα (Sano et al., 2013) or ERβ had a minimal effect on the expression of aggressive behavior. Because gonadally intact male mice were used as experimental animals in both studies, signaling of endogenous testosterone remained unaffected other than ERα or ERβ in the targeted area. Although it is possible that in the fully developed adult brain, two types of ERs may compensate functions of knocked down ER gene, it is likely that ER in the MPOA may not be involved in the regulation of aggressive behavior by activational action of testosterone in adult male mice.

### Role of ERβ in the MeA in the regulation of sexual and aggressive behavior

Prepubertal treatment with AAV-shERβ did not alter sexual and aggressive behavior in adulthood (Experiment 1). As expected from this finding, mice treated with AAV-shERβ in adult showed equivalent levels of sexual and aggressive behavior as control mice (Experiment 3). These results suggest that ERβ may not be responsible for either organizational or activational action of testosterone in the MeA. However, because we used corncob bedding, which has been reported to contain phytoestrogens (Landeros et al., 2012; Trainor et al., 2013), it is still possible that regardless of AAV treatment, all mice used in the present study may have been exposed estrogenic stimulation enough to partially organize neural circuitry before AAV injection on P21. If this is the case, we cannot completely rule out a possibility of the involvement of ERβ in organizational action of testosterone in the MeA. This needs to be addressed in the future studies.

In site-specific ERα knockdown mice, it is also reported that ERα in the MeA may not be involved in activational action of testosterone in the regulation of sexual and aggressive behavior (Sano et al., 2013) although it is essential for organizational action (Sano et al., 2015). MeA is known to be one of the critical brain regions for the induction of male sexual and aggressive behaviors (Vochteloo and Koolhaas, 1987; Kondo, 1992; Newman, 1999). In this brain region, aromatization to estradiol is essential for facilitation of these behaviors by testosterone (Wood, 1996; Unger et al., 2015). The present findings in βERKD mice in conjunction with those in αERKD mice (Sano et al., 2013) suggest that estradiol stimulation through either ERα or ERβ may be sufficient for induction of sexual and aggressive behavior in male mice. In contrast, a recent study in male rats reported that stimulation of both ERα and ERβ might be necessary for induction of sexual behavior, because MeA site-specific implant of either ERα or ERβ-specific agonist failed to restore sexual behavior in gonadectomized male rats systemically injected with dihydrotestosterone (Russell et al., 2012). It should be noted, however, in this study any ERs other than those in the MeA were not activated.

Given the fact that ERβ silencing did not alter the expression of sexual and aggressive behaviors, we further examined the effects of AAV-shERβ treatment on sexual preference. The MeA receives direct projections from main and accessory olfactory systems and sends social information to the hypothalamic nuclei including the MPOA and VMN, which are involved in the performance of male sexual and aggressive behaviors (Baum, 2009). In male rats, local lesions in the MeA disrupted sexual preference of receptive female over nonreceptive female rats (Kondo and Sachs, 2002). Recently, it is also reported that MeA lesions in male rats disrupt preference of receptive over nonreceptive females without affecting preference of a receptive female over an intact male (Dhungel et al., 2011). In the present study, we found that lack of ERβ in the MeA eliminated sexual preference between receptive female and nonreceptive females tested by the PTFF paradigm. During sexual preference tests with the PTFM paradigm, on the other hand, both βERKD and control groups showed clear preference toward a receptive female mouse over a gonadally intact male. Using soiled beddings as stimuli, it is also reported that global ERβ knockout male mice show normal preference toward receptive female odor over intact male odor (Kudwa et al., 2005). Collectively, these findings suggest that ERβ in the MeA is necessary for discrimination of receptivity states of female mice, but not discrimination of females from males. Along this line, it should also be noted that in both PTFF and PTFM tests, total SI duration exhibited toward two stimulus mice was not different between two treatment groups. This finding suggests that ERβ knockdown in the MeA did not affect sexual motivation of male mice, consistent with the fact that βERKD mice in the present study showed comparable number of mounts and intromissions as control mice during sexual behavior tests, in which only a receptive female mouse was presented. To this end, it is interesting to examine how βERKD mice may respond if receptive and nonreceptive female mice are simultaneously presented in sexual behavior tests.

## Conclusion

The present study provides evidence suggesting pubertal organizational action of ERβ in the MPOA for full masculinization of the neural network for male aggressive behavior. In the MeA, ERβ may be involved in information processing about receptivity states of female mice. Collectively, our results suggest that ERβ in the MPOA and MeA are involved in the regulation of male social behaviors in a manner different from that of ERα. In future studies, it is necessary to determine the role played by ERβ expressed in other brain sites in the social behavior network, including the bed nucleus of stria terminalis, lateral septum, and dorsal raphe nucleus.

## Synthesis

The decision was a result of the Reviewing Editor Cheryl Sisk and the peer reviewers coming together and discussing their recommendations until a consensus was reached. A fact-based synthesis statement explaining their decision and outlining what is needed to prepare a revision is listed below. The following reviewers agreed to reveal their identity: Robert Handa, Michael Steinman

This manuscript examines the role of estrogen receptor beta expression by neurons in the medial preoptic area and medial amygdala on male reproductive and aggressive behaviors. The authors have used RNAi to site-specifically knock down ERbeta using AAV. The authors importantly show that when ERbeta is knocked down in the MPOA of the prepubertal male they see decreases in aggressive bouts when tested in adulthood. This did not occur when MPOA ERbeta was knocked down in adulthood. In contrast, knockdown of ERbeta in the MeA had no affect prepubertally or in adulthood on either behavioral category, but it did impair the males' ability to determine estrous state of females. The studies use an innovative approach to identify the function of ERbeta in different brain regions. These findings are important and valuable for understanding organizational vs. activation effects of ER-beta on behavior. Overall the studies are well done, however there are a few concerns that should be addressed. In particular, the authors do not provide sufficient information to convincingly prove ER-beta knockdown and this should be addressed before this manuscript is suitable for publication is eNeuro.

1. The authors utilize a double immunocytochemical approach to determine that ERbeta ir is absent from GFP -ir cells, the GFP ir being indicative of neurons that have been infected with AAV. The authors could do more to convincingly demonstrate knockdown of ER beta, especially in view of the fact that there is a lot of null data (which is fine-overall the data make sense together but more information confirming knockdown would be very helpful).
  - There should be more information provided about the specificity of the antibody that was used. As the authors may be aware, there are currently no good antibodies for ERbeta to use for immunocytochemistry. The specificity of commercially available ER-beta antibodies is controversial and at a minimum they are known to be finicky. Although the original Zymed antibody was useful, this antibody has not been on the market for many years and lots beyond the original lot were not specific. Hence, additional information about the antiserum and the specificity of the lot used must be provided. A western blot or at a minimum a preabsorption study would be useful, especially if you preadsorbed with ER-alpha antigen to show that did not quench the antibody.
  - It is essential to present a graph or table showing a comparison of ER-beta in control versus knockdown animals for each experiment (pre- and post-pubertal) and each brain region. It is difficult to evaluate extent of knockdown using a single photomicrograph. Of importance, it appears from the pictures presented that all of the ERbeta is gone in MPOA and MeA, yet not all of the neurons in the field are infected with the AAV. In order for this to occur the AAV would have to be selecting for ERbeta expressing neurons, but this should not be the case. A better quantification of ERbeta knockdown should be provided for all brain regions and for all studies and more representative micrographs should be provided.
2. The authors inject AAV shortly after weaning, but only check ERbeta expression after behavioral testing. Do they know how soon after AAV injection that ERbeta was reduced in these brain regions? In addition, please include an image showing the extent of viral spread using GFP staining.
3. Have the authors determined that plasma testosterone levels in all groups are similar?
4. Have the author's tried analyzing the different aggressive behaviors separately? It' s very common for specific sub behaviors in RI to be affected by treatments. Knowing if there is a specific behavior or behaviors that drive the effect would aid interpretation.
5. The mice are raised on corncob bedding which contains phytoestrogens. Prepubescent mice may have experienced ER-beta agonism prior to the experimenters' knockdown manipulation and so this may have partially organized the circuit and affected results. This should be addressed in the discussion.
